# Acute effects of the sigma-2 receptor agonist siramesine on lysosomal and extra-lysosomal proteolytic systems in lens epithelial cells

**Published:** 2010-05-08

**Authors:** S. Jonhede, A. Petersen, M. Zetterberg, J-O. Karlsson

**Affiliations:** 1 Institute of Biomedicine, Department of Medical Chemistry and Cell Biology, University of Gothenburg, Gothenburg, Sweden; 2 Department of Clinical Neuroscience and Rehabilitation/Ophthalmology, Institute of Neuroscience and Physiology, The Sahlgrenska Academy, University of Gothenburg, Gothenburg, Sweden

## Abstract

**Purpose:**

The aim of the present study was to examine the effects of the sigma-2 receptor agonist, siramesine, on morphology, growth, cell death, lysosomal function, and effects on extra-lysosomal proteolytic systems in human lens epithelial cells.

**Methods:**

Human lens epithelial cells in culture were exposed to siramesine and examined for morphological changes using Nomarski optics or calcein. Lysosomes were evaluated using acridine orange and Magic Red (RR-cresyl violet). Nuclear morphology was studied using Hoechst 33342 and propidium iodide. Enzymatic activities in living cells or cell lysates were studied using fluorogenic substrates.

**Results:**

Siramesine at low concentrations increased the cytoplasmic proteolytic activity of the proteasome and the calpain system. Effects were also observed with respect to lysosomal morphology, acidity and function. In addition, activation of caspase-3 and the appearance of nuclei with an apoptotic morphology was found.

**Conclusions:**

Siramesine at low concentrations affects lens epithelial cells with perturbations of the major proteolytic systems and lysosomal morphology, resulting in caspase activation and cell death. Siramesine may be a possible substance for the treatment or prevention of posterior capsular opacification (PCO).

## Introduction

The sigma-2 receptor has been found in rapidly proliferating cells, including several human and rodent tumor cell lines [1,2], and it has been used as a marker for proliferation in human breast tumors [3]. Agonists to the sigma-2 receptor have antiproliferative and cytotoxic effects [4,5] and have been reported to give a caspase-independent cell death in tumor cells [6-8]. Sigma-2 receptor agonists have also been reported to affect Ca^2+^-release from the endoplasmic reticulum [9] and the inward rectifying K^+^ channels in the heart [10]. The subcellular localization of the receptor probably includes lysosomes, mitochondria, endoplasmic reticulum, and the plasma membrane [11]. The endogenous ligand(s) to the receptor is not known but some data suggest that the ligand(s) is internalized, in part, by the endocytotic pathway [11]. It has also been suggested that the sigma-2 receptor may be a histone binding protein [5]. An intriguing possibility is that siramesine exerts its effects by binding to phosphatidic acid on the bilayer surface [12]. This lipid may have a central role as a secondary messenger in many cellular functions.

Siramesine (Lu28–179; 1´-(4-(1-(4-fluorphenyl)-1H-indol-3-yl))butan-1-yl)spiro(isobenzofuran-1(3H),4´-piperidine) is a selective sigma-2 receptor agonist [13,14] which has been examined in detail by Jäättelä et al. [7,15]. Their results indicate a preferential effect on the lysosome with destabilization, enzyme leakage, oxidative stress, and accumulation of autophagosomes. These changes were followed by a caspase-independent apoptosis.

Whereas sigma-2 receptors appear to be proapoptotic, there are also sigma-1 receptors; these in contrast are antiapoptotic. Sigma-1 receptor antagonists have been shown to inhibit proliferation in colon and mammary cancer cell lines, which has lead to the development of potentially new anti-cancer drugs [16,17]. The expression of sigma-1 receptors, as well as sigma-1 receptor antagonist-induced apoptosis, has been demonstrated in human lens epithelial cells [18]. To our knowledge however, there has been no investigation of sigma-2 receptor-related effects in lens epithelial cells.

Posterior capsular opacification (PCO) is the most common complication after cataract surgery, resulting from proliferation of residual lens epithelial cells in the lens capsule. We are interested in the possibility of using the sigma-2 receptor agonist siramesine as a drug for inhibiting growth of lens epithelial cells to prevent the development of PCO. A significant advantage of this drug is that clinical trials have been conducted on siramesine for the treatment of anxiety and depression. The results from these trials show that siramesine is both non-toxic and well tolerated [19].

Experiments were thus performed to study the effects of the sigma-2 receptor agonist siramesine on markers for apoptosis and proteolytic activity in cultures of human lens epithelial cells (HLEC).

## Methods

### Materials

Human lens epithelial cells were obtained from lenses during cataract surgery at the Eye Clinic, Sahlgrenska University Hospital, Mölndal, Sweden, after informed consent. The collection was approved by the Regional Ethics Committee of Göteborg and the Declaration of Helsinki was followed.

The cell culture medium, RPMI-1640, and its additives fetal bovine serum (FBS), penicillin, amphotericin, as well as Hoechst 33342, propidium iodide, acridine orange, trypsin inhibitor, pepstatin, leupeptin, and PMSF were all acquired from Sigma Chemical (St. Louis, MO). Magic Red (cresyl violet) was manufactured by Immunochemistry Technologies (Bloomington, MN). The fluorogenic substrates Ac-Asp-Glu-Val-Asp-AMC (DEVD), Suc-Leu-Leu-Val-Tyr-7-amido-4-methylcoumarin (LLVY) and Z-Phe-Arg-AMC were from Bachem (Bubendorf, Switzerland) and the inhibitors lactacystin and calpeptin were from Calbiochem (San Diego, CA) and Novobiochem (La Jolla, CA), respectively. Calcein was from Molecular Probes (Eugene, OR) and Siramesine was kindly provided by Christian Thomsen, H. Lundbeck A/S, Valby, Denmark.

### Human lens epithelial cell culture and treatment

The human lens epithelium specimens, usually 5 mm in diameter, were placed into Eppendorf tubes, which contained culture medium (RPMI-1640) supplemented with 10% fetal calf serum, 100 U/ml penicillin, 0.1 mg/ml streptomycin, and 2 mM L-glutamine and 2,5 µg/ml amphotericin, immediately after surgery. The lens epithelium specimens were later transferred from the Eppendorf tubes to 24 well culture dishes (TPP, Trasadingen, Switzerland) in a humidified CO_2_ incubator at 37 °C to allow the capsule to attach to the bottom of the culture well. After one to two weeks, human lens epithelial cells (HLECs) on the capsules and cells that had migrated onto the bottom of the culture well were detached by trypsinization and seeded in cell culture flasks (75 cm^2^). At confluency, cells were subcultured by 0.25% trypsin/ EDTA treatment. For each experiment, human lens epithelial cells from one individual, passage 6–10, were grown on white 96 well plates with transparent bottom (Costar Corp., Cambridge, MA) in supplemented RPMI-1640 media. The plates were incubated in a humidified CO_2_ incubator at 37 °C until desired confluency of the cells was obtained (approximately 10^5^ cells per well). Siramesine was diluted in DMSO to make a 30 mM stock solution, which was then used when diluting to reach desired concentrations. All equipment, such as pipet tips, tubes, and plates was coated with serum-containing media before addition of siramesine to avoid binding of the hydrophobic compound to the plastic surfaces. All experiments were performed at least three times. When repeated, another cell line with a similar phenotype and growth pattern was chosen. Altogether 3 different cell lines were used. All cells derived from randomly selected age-related cataractous lenses. No information about the age of the donor or the cataract type was obtained. Control HLECs were exposed to solvent at relevant concentrations.

### Confocal microscopy

Morphology of cells incubated with siramesine was examined using a confocal microscope equipped with an Argon, HeNe and diode laser (Eclipse TE300; Nikon, Tokyo, Japan). HLEC were grown on cover glass precoated with collagen. Siramesine was added 1–3 h before the dyes. Nuclear morphology was viewed using Hoechst 33342 (final concentration was18.7 µM) or propidium iodide (final concentration was 15 µM). General cell morphology was evaluated using Nomarski optics or by staining with calcein (final concentration was 5 µM). Changes in lysosomal acidity were monitored using acridine orange (final concentration was 2 µg/ml) and lysosomal morphology was visualized using Magic Red (cresyl violet), at a concentration established by the manufacturer’s protocol (Immunochemistry Technologies, LLC, Bloomingdale, MN). All images were observed using Nikon Plan–Apo 20x or Plan-Apo 60x (water immersion) objectives.

### Apoptosis and mitosis

To examine apoptosis and mitosis, HLEC were grown on 24-well plastic plates and when desired confluence (~80%) was reached the cells were incubated with siramesine diluted in EBSS (120 mM NaCl, 5.4 mM KCl, 0.81 mM MgSO_4_, 1 mM NaH_2_PO_4_, 5,5 mM D-Glucose, 0.2 mM CaCl_2_, 25 mM Hepes, 100 U/ml penicillin, 0.1 mg/ml streptomycin, and 2 mM L-glutamine). The siramesine solutions were added at varying time intervals. The cells were then stained with Hoechst and fixed before counting. Examining nuclear morphology and counting the number of apoptotic and mitotic nuclei determined the extent of apoptosis and mitosis. Apoptotic nuclei were recognized by chromatin condensation and disintegration of the nucleus into apoptotic bodies. Mitotic nuclei, on the other hand, were identified by the chromosomal positional changes that take place during mitosis. The percentage of cells in either stage was determined in each well. At least 300 cells were evaluated in each well and each time point was tested in triplets.

### Assay of caspase activity

Caspase-3 activity was measured on 96-well plates of cell lysates. The cells were allowed to grow to complete confluence. Varying concentrations of siramesine diluted in EBSS were added to the cells at different time intervals during which the plate was placed in 37 °C. After desired incubation time, the buffer was removed and the plate was frozen at −52 °C. The plate was thawed and the cells were lysed in 0.1% CHAPS buffer (50 mM Tris-HCl, 100 mM NaCl, 5 mM EDTA, 1 mM EGTA, 3 mM NaN_3_, pH 7.3) with the addition of the following inhibitors; trypsin inhibitor (final concentration was 5 µg/ml), pepstatin (final concentration was 0.5 µg/ml), leupeptin (final concentration was 1.25 µg/ml) and PMSF (final concentration was 0.5 mM). The cells were incubated with the lysate buffer and inhibitors for 30 min at room temperature before an equivalent volume of substrate solution was added to each well and the caspase-3 activity was measured continuously during 2 h in a microplate spectrofluorometer (SPECTRAmax GEMINI; Molecular Devices, Sunnydale CA) at an excitation of 380 nm and an emission of 460 nm. The substrate solution contained DEVD (final concentration was 25 µM; Ac-Asp-Glu-Val-Asp-AMC) and DTT (final concentration was 4 mM) diluted in buffer (50 mM Tris-HCl, 100 mM NaCl, 5 mM EDTA, 1 mM EGTA, 3 mM NaN_3_, pH 7.3) [20]. V_max_ was calculated from fluorescence data using SOFTmax PRO Version 4.8 software (Molecular Devices, Sunnyvale, CA).

### Assay of proteolytic activity in living cells

Proteolytic activity was measured in unlysed cells using the synthetic peptide LLVY (Suc-Leu-Leu-Val-Tyr-AMC). A 40 mM stock solution of LLVY was prepared in 100% DMSO. The substrate was diluted to 200 µM in EBSS buffer and 50 µl was added to each well to yield a final concentration of 50 µM. Prior to the addition of the substrate solution, the cells were incubated with varying concentrations of siramesine. The plate was placed in the spectrofluorometer and the V_max_ was calculated from continuous measurement of the fluorogenic cleavage product AMC. The proteolytic activity was also measured after addition of known inhibitors of proteases. The cells were grown on plates as above after which siramesine (30 µM) was added as well as 10 µM and 50 µM, respectively, of the inhibitors lactacystin and calpeptin. Controls with 0.1% DMSO were included on the plate. After 30 min of incubation with inhibitors and siramesine at 37 °C the LLVY solution was added and the fluorescence of the cleavage product, AMC, was measured continuously in the spectrofluorometer at an excitation of 380 nm and an emission of 460 nm during a period of 2 h at 37 °C to determine V_max_.

### Cathepsin assay

Cathepsin activity was measured both in living cells as well as in cell lysates made from HLEC. To determine the activity in living cells, HLEC were grown on 96-well plates and when confluent incubated with concentrations between 0 µM and 30 µM of siramesine for 1 h. After the incubation, the Cathepsin B substrate, Z-Phe-Arg-AMC was added at a final concentration of 50 µM. The plate was then placed in the spectrofluorometer and the fluorescence of the cleavage product was measured at an excitation of 380 nm and an emission of 460 nm continuously for 2 h at 37 °C. V_max_ was then calculated using SOFTmax® PRO as software. The cathepsin activity of lysed cells was measured using HLEC lysates. Lysates were prepared by trypsinating and condensing one flask of cells into 1 ml of lysis buffer (100 mM Na-Acetate, 50 mM NaCl, 1%Triton, 1 mM EDTA, pH 5.5) and freezing (−152 °C) over night. The assay was run on white 96-well plates where lysate was mixed with siramesine diluted in reaction buffer (100 mM NaAcetate, 50 mM NaCl, 2 mM DTT, pH 5.5) to yield final concentrations during measurement between 0 and 30 µM. The mixture was allowed to incubate on ice for one hour before substrate (Z-Phe-Arg-AMC) was added to a final concentration of 50 µM. The fluorescence was measured as described above.

### Statistics

ANOVA with Dunett as post-hoc was used for experiments with multiple data and unpaired *t*-tests were used for experimental results with only two comparative groups. Means±SEM are shown where n≥3. A p-value of <0.05 was considered significant. SPSS, version 13.0 for Mac (SPSS Inc., Chicago, IL) was used as statistic software.

## Results

### Siramesine induces rapid changes in cellular morphology

**Figure 1:**
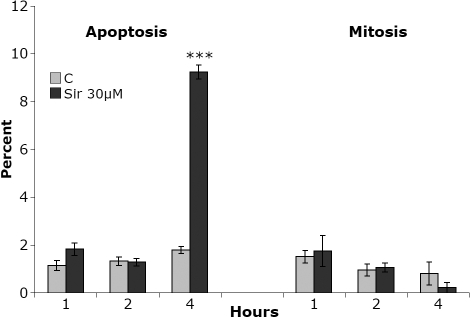
Apoptosis and mitosis in human lens epithelial cells (HLEC) following incubation with siramesine. Cells were stained with Hoechst and the nuclear morphology was examined by confocal microscopy. The diagram shows the average percentage of cells in three independent wells displaying apoptotic and mitotic nuclei after 1–4 h of incubation with siramesine or solvent (0.1% DMSO; C). A clear proapoptotic effect of siramesine was observed already after 4h. A tendency for a reduced mitotic activity was observed at this time. Results are displayed with mean±SEM, p<0.001 (***). The experiment was repeated three times with similar results.

**Figure 2:**
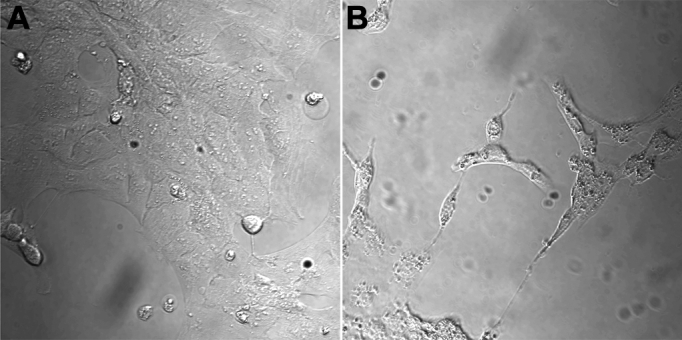
Morphological changes of human lens epithelial cells (HLEC) with perinuclear vesicular structures after siramesine treatment as revealed by DIC optics. The control cells were exposed to solvent, 0.1% DMSO (**A**) or 15 µM of siramesine for 2.5 h at 37 °C (**B**).

**Figure 3:**
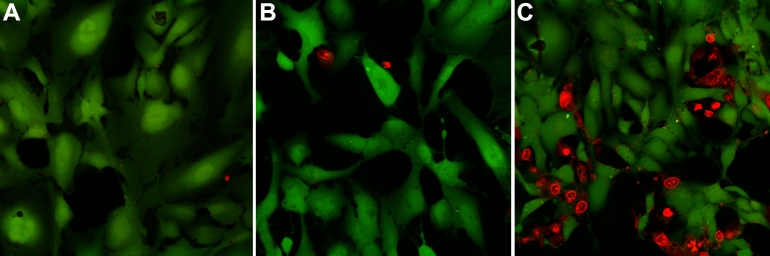
Decreased viability after siramesine treatment as shown by the increased nuclear propidium iodide staining. Human lens epithelial cells (HLEC) were exposed to solvent (0.05% DMSO; **A**), 5 µM siramesine (**B**) and 15 µM siramesine (**C**) for 3 h and stained with propidium iodide (red) and calcein (green).

Human lens epithelial cells in culture responded to siramesine treatment with a relatively rapid (within 4 h) change of nuclear morphology, such as condensation and fragmentation of chromatin (Figure 1). No significant changes in mitotic index were observed, although a tendency to decrease was found in most experiments (Figure 1). Vesicular structures, usually with a perinuclear localization, were visible approximately 20 min to 2 h following administration of siramesine at concentrations from 10 to 30 µM (Figure 2A,B). Cytoplasmic staining with calcein revealed significant siramesine-induced changes of cellular morphology after 2 to 3 h with 5–15 µM siramesine (Figure 3). Many cells appeared shrunken and an increased number of nuclei stained with propidium iodide were observed, demonstrating irreversible membrane dysfunction and cell death.

### Siramesine activates caspases

**Figure 4:**
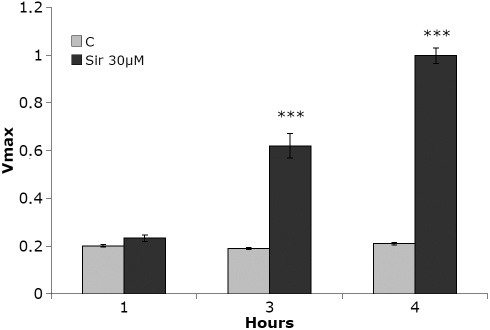
Caspase 3 activity in cells after addition of siramesine. Human lens epithelial cells (HLEC) displayed a substantial increase in caspase 3 activity 3–4 h after exposure to 30 µM siramesine. The experiment was repeated four times and the diagram shows representative results where n=3. Mean±SEM is shown. p<0.001 (***).

Siramesine at a concentration of 30 µM induced a significant increase in caspase-3 activity from 3 h up to 6 h after exposure (Figure 4). The increase in caspase activity at 4 h was approximately fivefold and corresponded closely to the observed changes of nuclear morphology. During this time the percentage of nuclei with apoptotic morphology increased from 1.9 percent to 9 percent.

### Siramesine increases cytoplasmic protease activity

**Figure 5:**
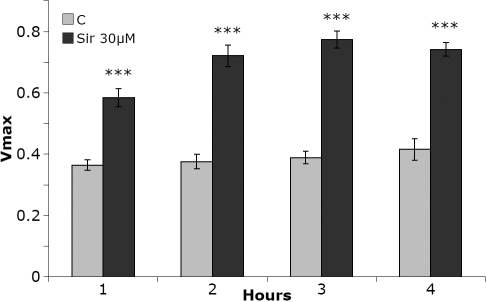
Increased proteolytic activity in human lens epithelial cells (HLEC) using LLVY. The assay was performed on intact cells and a significant siramesine dependant increase was observed at all times examined.  The rate of substrate cleavage was measured after incubating the cells with 30 µM siramesine for 1–4 h. The figure shows the average of n=3. Mean±SEM is shown. p<0.001 (***). Three independent experiments were performed with similar results.

**Figure 6:**
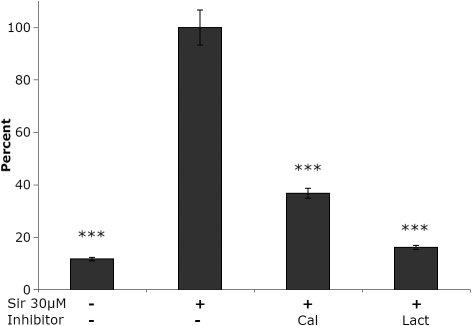
Characterization of extralysosomal proteolytic activity. The results indicates a marked effect of siramesine on both the proteasome and calpain proteolytic systems. Reduction of proteolytic activity in human lens epithelial cells (HLEC) incubated with 30 µM siramesine (100 percent on the y-axis) after addition of calpeptin (Cal) or lactacystin (Lact). Proteolysis was measured with the synthetic peptide substrate LLVY, which can be cleaved both by the proteasome and by calpain. Formation of the cleavage product was measured continuously and the effects of the proteasome inhibitor lactacystin and the calpain-inhibitior calpeptin are shown relative to that of the proteolytic activity in cells exposed to 30 µM siramesine without inhibitor. The experiment was repeated three times. n=16. Mean±SEM is shown. p<0.001 (***).

In the intact cells siramesine rapidly caused an increased activity of important cytoplasmic proteases (Figure 5). The substrate used (LLVY) have previously been shown to be a good substrate both for calpains and the proteasome [21]. The major part of the increased degradation of the LLVY-substrate was probably due to proteasome activity as indicated by the marked effect of the specific proteasome inhibitor lactacystin (Figure 6). Addition of the calpain inhibitor calpeptin also caused a significant change in LLVY-degrading activity, but did not lead to a complete inhibition (Figure 6).

### Siramesine affects lysosomal activity

**Figure 7:**
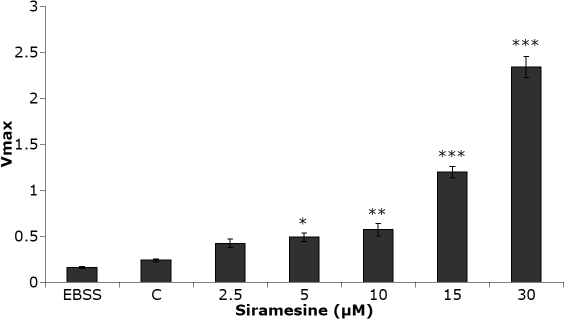
Cathepsin activity in human lens epithelial cells (HLEC) after incubation with siramesine. The activity of lysosomal cathepsins was clearly sensitive to siramesine treatment. The diagram shows the dose-dependent increase in proteolytic activity of cathepsin after addition of siramesine 1 h before substrate addition. V_max_ is shown with mean±SEM where n=8 and significance levels p<0.05 (*), p<0.01 (**) and p<0.001 (***). Three independent experiments were performed with similar results.

**Figure 8:**
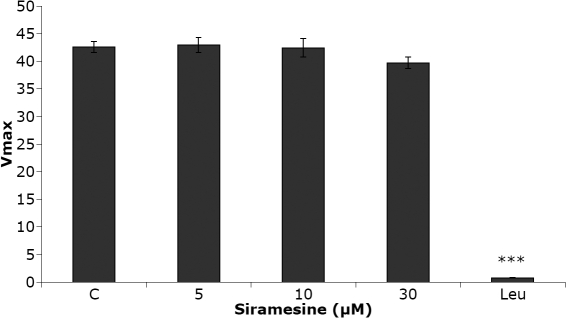
No effect of siramesine on cathepsin activity in cell lysates from human lens epithelial cells (HLEC). The enzymatic activity measured in cell lysates with final concentrations of siramesine between 0 and 30 µM at a pH of 5.5. Leupeptin (50 µM), a known Cathepsin B inhibitor, was used as a negative control and displayed a significant drop in activity. The diagram shows V_max_ at each concentration of siramesine where n=3. Mean±SEM is shown. p<0.001 (***). The experiment was repeated twice with similar results.

The ability of intact lens epithelial cells to take up and degrade the slightly hydrophobic cathepsin substrate (FR-AMC) was clearly increased after siramesine treatment (Figure 7). The increased activity, probably mostly cathepsin B activity, was significant already after 1 h of incubation with 5 µM of siramesine. However, lysosomal activity assayed at pH 5.5 in vitro, using lysates of lens epithelial cells, revealed no direct effects of siramesine on cathepsin activity at concentrations between 0 and 30 µM (Figure 8).

### Siramesine affects lysosomal morphology and acidity

**Figure 9:**
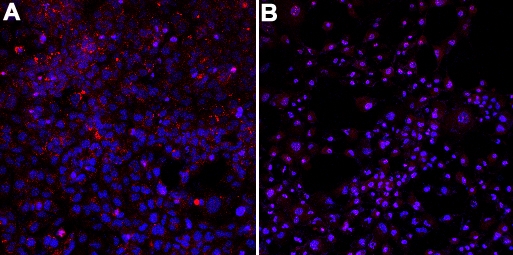
Lysosomal acidity is decreased after siramesine treatment. As shown by the marked decrease of red staining indicating the alkalinization of the lysosomal pH. The sample was excited with the UV–laser. Human lens epithelial cells (HLEC) were stained with acridine orange (red) and Hoechst (blue) after incubation with 10 µM siramesine (**B**) and solvent, 1% DMSO (**A**). One of two experiments is shown.

**Figure 10:**
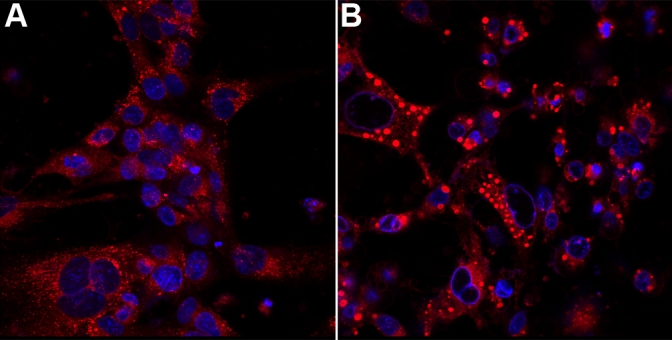
Lysosomal morphology is changed after siramesine treatment. Note the large and heavily stained lysosomal structures in the treated cells. Human lens epithelial cells (HLEC) was exposed to 30 µM of siramesine (**B**) or solvent (**A**) for 3 h at 37 °C before addition of the fluorogenic cathepsin B substrate Magic Red (RR-cresyl violet; red). Cell nuclei are stained with Hoechst (blue). Confocal microscopy was performed 30 min after addition of the substrate. More than three independent experiments were performed with similar results.

Acridine orange staining of HLEC revealed that the lysosomal acidity was severly reduced 4 h after administration of 10 µM siramesine. (Figure 9A,B). In an attempt to visualize lysosomal cathepsin activity in cultured cells in vivo we used a cell-permeable substrate specific for cathepsin, Magic Red (RR-cresyl violet) that upon cleavage will form a fluorescent product trapped in the lysosome. Our data indicated that the fluorescent reaction product (cresyl violet) accumulated in vesicular structures that may correspond to lysosomes (Figure 10). The cresyl violet stained material appeared to form larger perinuclear structures after administration of siramesine as compared to cells exposed to solvent only.

## Discussion

Posterior capsular opacification (PCO) is the result of proliferation of residual lens epithelial cells after cataract surgery. Epithelial cells on the anterior lens capsule proliferate and migrate posteriorly, causing opacification and wrinkling of the posterior lens capsule, eventually leading to impaired vision. Actions to remove residual lens epithelial cells at cataract surgery or to prevent growth of these cells have not been entirely successful [22], although design of the intraocular lens (IOL) prosthesis have proved to be of importance [23]. A substance that affects the proliferation and death of lens epithelial cells without causing other toxic effects in the body would be a possible candidate to treat patients with PCO. Siramesine may act both as a cytoprotective agent that induce the formation of autophagosomes and (at a later stage) the promotion of cell death [24]. In the lens epithelium the drug may possibly play a role in the delicate regulation of cell proliferation, differentiation and cell death in the anterior and equatorial region of the lens.

Our results clearly show that human lens epithelial cells in culture are sensitive to relatively low concentrations of siramesine. Morphological and proteolytical changes suggestive of apoptosis were seen 3 to 4 h after administration of 30 µM siramesine. The sensitivity was comparable to that observed in many human and rodent tumor cell lines [7]. In contrast to previous studies [6-8], the apoptosis shown in this study was clearly caspase-associated.

Very early changes of cellular morphology were observed after siramesine exposure. These changes in the perinuclear region may correspond to lysosomal structures and our experiments with a cell-permeable lysosomal-specific protease substrate indicated that the reaction product accumulated in larger lysosomal structures as compared to that of cells not treated with siramesine, which exhibited much smaller lysosomal structures.

Changes in the activity of lysosomal enzymes indicated increased activity already at 5 µM of siramesine. If these results reflect a real increase in lysosomal protease activity or an increased availability of the substrate to the cytoplasm and/or the lysosome interior remains to be studied. Siramesine had no effect on cathepsin activity in lysates of lens epithelial cells. We used acridine orange to stain the acidic compartment of the cell, roughly corresponding to the lysosomes. Our results clearly showed that the staining decreased very early and was heavily reduced a few hours after siramesine treatment. This indicated that the lysosomal membrane or proton pump, at this time, was compromised and that the acidic milieu had disappeared.

Examination of the major cytoplasmic (extra-lysosomal) proteolytic systems indicated that an increased proteolytic activity could be observed already 1 h after siramesine treatment. Our findings thus clearly indicated a marked sensitivity of HLEC to siramesine. Using inhibitors of these proteolytic systems, we could demonstrate that a specific proteasome inhibitor could abolish most of the activity. A significant effect was also observed when inhibiting the calpain system. The cytotoxic effects induced by siramisine in HLEC may thus be initiated by perturbations of the major proteolytic systems, the proteasome-ubiquitin system and the calpain family. The mechanism behind the high sensitivity to siramesine in lens epithelial and tumor cells, as opposed to other cell types, is not known. We have not observed any differences in siramesine sensitivity between confluent and rapidly proliferating lens epithelial cells (data not shown). It has earlier been suggested that tumor cells have more of the sigma-2 receptor than normal cells but others have speculated upon differences in the signal transduction process [1,7]. Another possibility could be a variation in the expression of phosphatidic acid at the cell surface between different cell types.

Posterior capsular opacification (PCO) is the most frequent complication to cataract surgery, with reported incidence numbers of 20%–40% [22]. Even though PCO can be treated successfully by posterior capsulotomy using Nd:YAG laser, this treatment has potential risks such as development of macular edema and retinal detachment [25,26]. An agent that can reduce the growth of residual lens epithelial cells without exerting toxic effects on other cell types or on neighboring tissues, is an attractive idea. However it still remains to be elucidated if siramesine, in an intact organism, is selective to lens epithelial cells as compared to retinal cells. Animal studies are required to resolve this issue.
